# Assessing the Impact of Bycatch on Dolphin Populations: The Case of the Common Dolphin in the Eastern North Atlantic

**DOI:** 10.1371/journal.pone.0032615

**Published:** 2012-02-29

**Authors:** Laura Mannocci, Willy Dabin, Emmanuelle Augeraud-Véron, Jean-François Dupuy, Christophe Barbraud, Vincent Ridoux

**Affiliations:** 1 Littoral, Environnement et Sociétés, UMR 7266, Université de La Rochelle-CNRS, La Rochelle, France; 2 Observatoire Pelagis, UMS 3462, Université de La Rochelle-CNRS, La Rochelle, France; 3 Mathématiques Image et Applications, Université de La Rochelle, La Rochelle, France; 4 Centre d'Etudes Biologiques de Chizé, UPR 1934, CNRS, Villiers en Bois, France; Monash University, Australia

## Abstract

Fisheries interactions have been implicated in the decline of many marine vertebrates worldwide. In the eastern North Atlantic, at least 1000 common dolphins (*Delphinus delphis*) are bycaught each year, particularly in pelagic pair-trawls. We have assessed the resulting impact of bycatch on this population using a demographic modeling approach. We relied on a sample of females stranded along the French Atlantic and western Channel coasts. Strandings represent an extensive source of demographic information to monitor our study population. Necropsy analysis provided an estimate of individual age and reproductive state. Then we estimated effective survivorship (including natural and human-induced mortality), age at first reproduction and pregnancy rates. Reproductive parameters were consistent with literature, but effective survivorship was unexpectedly low. Demographic parameters were then used as inputs in two models. A constant parameter matrix proposed an effective growth rate of −5.5±0.5%, corresponding to the current situation (including bycatch mortality). Subsequently, deterministic projections suggested that the population would be reduced to 20% of its current size in 30 years and would be extinct in 100 years. The demographic invariant model suggested a maximum growth rate of +4.5±0.09%, corresponding to the optimal demographic situation. Then, a risk analysis incorporating Potential Biological Removal (PBR), based on two plausible scenarii for stock structure suggested that bycatch level was unsustainable for the neritic population of the Bay of Biscay under a two-stock scenario. In depth assessment of stock structure and improved observer programs to provide scientifically robust bycatch estimates are needed. Effective conservation measures would be reducing bycatch to less than 50% of the current level in the neritic stock to reach PBR. Our approach provided indicators of the status and trajectory of the common dolphin population in the eastern North Atlantic and therefore proved to be a valuable tool for management, applicable to other dolphin populations.

## Introduction

Fisheries bycatch has been implicated in the declines of marine vertebrates worldwide, such as sea turtles, seabirds and marine mammals [1]. In addition, their large size, high trophic level and vast habitat expose these species to many other anthropogenic pressures such as direct exploitation, competition for resources, habitat modification or chemical pollution. Because of their late maturation and long life-span, small cetaceans are very sensitive to human-induced additional mortality and have a limited capacity for population recovery.

In the eastern North Atlantic (ENA), short-beaked common dolphin (*Delphinus delphis*) has been reported as bycatch in several fisheries, including tuna driftnet fishery [2], pelagic pair-trawl fishery [3], [4], gillnet fishery [5] and set gillnet fishery [6]. Onboard observer programs dedicated to estimate bycatch in different fisheries under EU regulation No 812/2004 (26 April 2004) [7] suggest that a minimum of 1000 common dolphins are incidentally caught every year in the ENA [8]. Although this regulation is an important step forward to better assess bycatch in European fisheries, it also has inherent limitations as member states are currently only required to monitor cetacean bycatch on board vessels with an overall length of 15 m or more, albeit vessels under 15 m long represent a vast majority of fishing fleets in all EU countries [9].

It is unclear whether the common dolphin population consists of a single stock or should be separated into two or more populations in the ENA. Genetic studies did not show differentiation of common dolphins in the ENA, suggesting a single genetic stock in this region [10]. However, ecological approaches, analyzing heavy metals [11], [12] and stable isotopes [12], indicate a separation of the common dolphins living on the continental shelf (neritic stock) from those living offshore (oceanic stock). This conforms to US Marine Mammal stock assessments where definition of stock is based on the smallest divisible units which are biologically reasonable and practical from a management perspective [13]. In our stock scenarii definition we follow the International Whaling Commission (2010) conclusion that more information is needed to establish common dolphin population structure from ecological tracers and think that until this issue is fully resolved the one-stock and two-stock scenarii could be use to bracket the range of population structure [8].

Aerial and shipborne surveys yielded an estimated population size of 63 366 (CV = 0.46) individuals for the European continental shelf (SCANS-2 census; [14]) and 116 709 (CV = 0.34) individuals for the offshore waters (CODA census across the Atlantic Exclusive Economic Zone of United Kingdom, Ireland, France and Spain; [15]). When combined, these censuses provided an estimate of 167 216 (CV = 0.25) individuals for most of European Atlantic waters [15], the offshore boundary of the area being solely defined on administrative consideration.

Two main approaches have been used to evaluate the impact of bycatch on marine megafauna. Several authors have developed population projections based on deterministic or stochastic matrix models [16]–[18]. These require estimates of age-specific survival and fertilities. Other studies, where limited demographic information is available, were based on the Potential Biological Removal procedure of the US Government (PBR; [19]), mostly for cetaceans [20], [21] and seabirds [22], [23]. PBR is the number of anthropogenic mortalities that allows the population to remain above its optimum sustainable level, situated between carrying capacity and maximum net productivity. PBR is calculated given an estimate of the population size, its maximal growth rate and a recovery factor [19].

Dolphin demographic parameters can be estimated directly from repeated observations of marked individuals [24], but such longitudinal studies are difficult for pelagic populations with broad distributions like the common dolphin in the ENA. Demographic parameters can also be estimated through the examination of biological samples provided by strandings or bycaught individuals (*e.g.* reproductive parameters; [25], [26]). However, there have been few attempts to estimate survivorship based on such age-at-death distributions. Strandings represent the most extensive source of demographic information to monitor our study population. Even if they do not result from a rigorous sampling scheme, stranding data can provide useful population indicators and can overcome certain bias inherent to more conventional and statistically robust monitoring methods. For example, all sources of anthropogenic mortality are susceptible to be reflected in strandings, whereas fisheries observer programs currently only monitor certain segments of fishing fleets.

Here we assessed the impact of bycatch on common dolphins in the eastern North Atlantic by using demographic modeling. We estimated effective survival and reproductive parameters based on a sample of stranded females for which reproductive state and age were known. We used these parameters as inputs in two demographic models and we incorporated deterministic population projections and risk analysis in order to examine management priorities for the population.

## Materials and Methods

### Available material and study area

**Figure 1:**
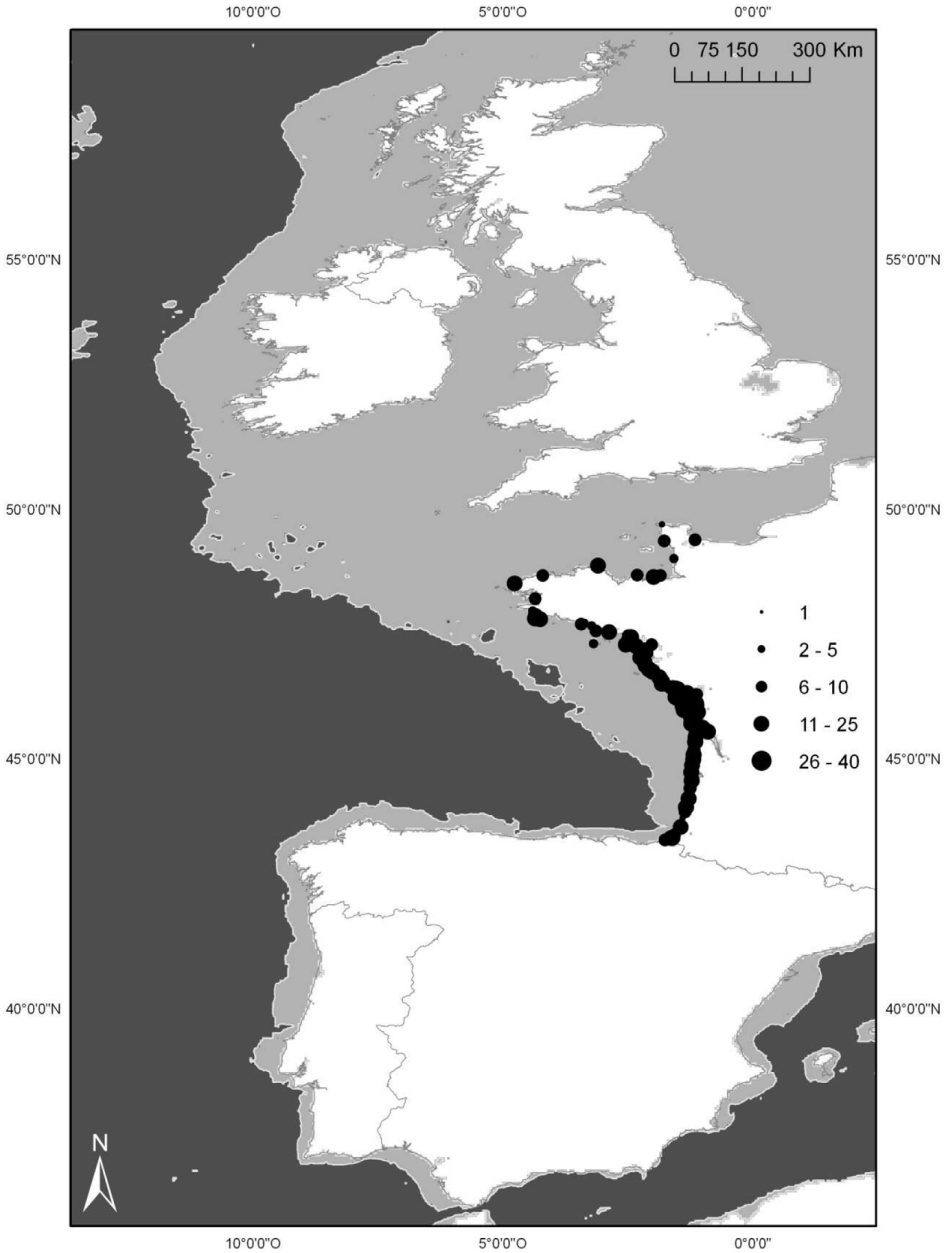
Study area and collection locations of female common dolphins (n = 406). The continental shelf and offshore waters are represented is light grey and dark grey respectively.

Our sample set comprised female short-beaked common dolphins (*D. delphis*) stranded on the French Atlantic and western Channel coasts from 1972 to 2006, collected by the French stranding network (Fig. 1). The vast majority of strandings occurred from January to March. Sex and gross health state were assessed by visual examination. A bycatch diagnosis was made when the individual had a good health state, showed signs of contact with fishing gear (skin lesions, skull fractures), evidence of hypoxia and other signs associated with damage during release from the fishing gear [27]. However, full diagnosis was only possible when lesions were not obliterated by *postmortem* autolysis. Other natural or anthropogenic causes of death with few external signs were more difficult to diagnose.

*Postmortem* examinations were carried out following a standard protocol [28]. Teeth were taken from the middle of the lower jaw. Age was determined by counting Growth Layer Groups (GLGs) in the dentine. Cross reading were carried out between laboratories involved in age estimation as part of the BIOCET EU program. We assumed common dolphin deposit a single GLG per year on the basis of calibration studies [29].

Entire reproductive tracts and mammary glands were collected. We assessed female reproductive state by examination of the reproductive tract following specific criteria [30]. Both ovaries were examined for the presence of *Corpora Albicantia* and (or) *Corpora Lutea*. The *Corpus Luteum* (*CL*) develops following the eruption of a mature follicle and persists as the endocrine gland of pregnancy if successful fertilization occurs. In the absence of fertilization, or after parturition, it degrades into a *Corpus Albicans* (*CA*). There are still questions about the persistence of *CA*s as visible ovarian scars in common dolphins [31], [32]. Females were categorized as immature when no structure was present on the ovaries (no *CA* or *CL*). They were defined as resting mature when they had at least one *CA* and showed no signs of gestation or lactation, as pregnant when a foetus or a *CL* associated with an expanded uterine horn were found and as lactating when milk was found in the mammary glands [33].

### Reproductive parameters

We defined age at sexual maturity (ASM) as the age at which 50% of the females are mature [34]. We estimated ASM by fitting a logistic regression to the proportion *p(x)* of mature females at age *x*:
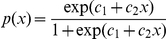
where *c_1_* and *c_2_* are estimated with the maximum likelihood procedure. ASM is given by *−c_1_/c_2_* [20], [26]. We described uncertainty in ASM by using non parametric bootstrap [35]. The maturity data were randomly re-sampled with replacement 1000 times, for each sample of the data the logistic regression was re-fitted and an estimate of ASM was obtained, generating a distribution of ASM. We estimated age at first reproduction (AFR) by assuming females conceived immediately after attainment of sexual maturity and adding an approximate gestation period to the ASM distribution [20], [36], [37]. Gestation period is approximately one year for common dolphins [26]. We obtained a distribution of AFR, described by its mean and standard deviation (sd).

Pregnancy rate was estimated by the ratio of pregnant females in the sample of mature females [25]. The reciprocal of pregnancy rate provided the calving interval. As numbers of females in 1-year age groups were small, we pooled data in 3-year age groups. We described uncertainty by treating each pregnancy rate as a binomial random variable [20], with the number of trials as the number of mature females and the probability of success as the pregnancy rate in each age group.

### Survival parameters

We estimated an effective survival from the age-at-death distribution of stranded common dolphins as it resulted from both natural and human-induced mortality. We fitted the Siler competing-risk model [38] to the age-at-death distribution. In this model, the total risk of mortality at a given age was expressed as the sum of a decreasing risk due to juvenile factors, an increasing risk due to senescent mortality factors and a constant risk affecting all age classes. Thus the probability of survivorship from birth to age *x* (*l(x)*) was expressed as:
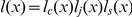
where:


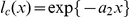
 represents the constant risk of mortality experienced by all age classes;


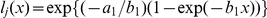
 represents the risk of mortality due to juvenile factors and


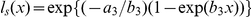
 represents the risk of mortality due to senescent factors;

The parameters (*a_1_,a_2_,a_3_,b_1_,b_3_*) are positive.

The likelihood for the parameters (*a_1_,a_2_,a_3_,b_1_,b_3_*) from the observed age-at-death distribution *(x_1_,x_2_,x_n_…)* (where *x_1_* is the age of female number 1, …, *x_n_* the age of female number *n*) is:
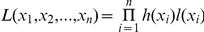
Where *l(x)* is the age-specific survivorship and *h(x)* is the hazard rate, calculated as −*d[ln(l(x))]/dx* [39]. The five parameters were estimated by maximizing the logarithm of this likelihood using a Newton-type algorithm [40].

### Demographic modeling

After estimating survival and reproductive parameters, we used them in two demographic models. First, we conducted deterministic population projections based on the effective growth rate estimated from a matrix model. It would reflect the current situation, given the additional mortality caused by bycatch. Then, we performed a risk analysis incorporating PBR, based on the maximal growth rate estimated from the demographic invariant model. It would reflect the optimal demographic situation. It is important to highlight that the two modeling approaches rely on different hypothesis concerning the demographic parameters, notably survival. We used the effective survival probabilities estimated from the Siler model to build the age-dependent matrix whereas we assumed a constant and optimal adult survival in the demographic invariant model.

#### Matrix model and deterministic population projections

We considered the following matrix population model:
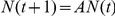

*w*here the vector *N* gives the number of individuals in each age class and the age-structured matrix *A* projects the population from *t* to *t+1* [41]. Demographic parameters estimated from strandings were combined into the matrix:
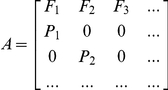
where *P_x_* are Siler age-specific survival probabilities calculated from the survivorships as *P(x) = l(x)/l(x-1)* and *F_x_* are fertilities, *F_x_ = m_x_P_x_*, where *m_x_* are female birth rates obtained by dividing pregnancy rates by two. This assumes a sex ratio of 1∶1 and that all observed pregnancies are carried to term.

We used the asymptotic matrix properties to estimate population effective growth rate λ, calculated as its dominant eigenvalue [41]. This process was repeated 1000 times to give a distribution of λ (described by its mean and standard deviation), incorporating uncertainty in AFR and pregnancy rates. In order to determine the relative impact of proportional changes in demographic parameters to λ, or their contribution to λ [42], we performed an elasticity analysis. We calculated the elasticity matrix E from the eigenvectors and coefficients of the mean matrix [41]. Then we summed the elasticities of λ to changes in *P_λ_* and *F_λ_* across age classes to obtain fertility, juvenile survival and adult survival elasticities [43].

Finally we performed deterministic projections using a 100-year projection period which is commonly taken for management purposes [19], [44].

Analyses were performed using R software and DemogR package.

#### Demographic invariant model and risk analysis

We considered a risk analysis where the risk is defined as the probability that annual bycatch exceed the PBR, defined as:
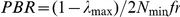
where *λ_max_* is the maximal growth rate, *N_min_* is the minimal population size, defined as the 20^th^ percentile of its sampling distribution assumed to be lognormal, and *fr* is a recovery factor ranging between 0.1 and 1 [19]. The recovery factor reflects the status of the stock and the perceived quality of the data. We set *fr* to 0.5, the value suggested for most healthy populations.

We estimated λ*_max_* from the demographic invariant model [22]. It allows the estimation of λ*_max_* knowing only estimates of age at first reproduction (*a*) and adult survival probability (*s*) for optimal conditions (that is without growth limiting factors). Assuming a constant fecundity and a constant adult survival after age at first reproduction, λ*_max_* is estimated as follows:
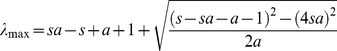



In practice population parameters may not be available for the species of interest and would rarely be available for optimal conditions [23]. We hypothesized that the age at first reproduction (AFR) estimated from strandings was valid under optimal demographic conditions. The age-at-death distribution derived from strandings allowed us to estimate effective survival. By causing additional mortality, bycatch is likely to limit population growth and therefore our survival estimate may not be representative of optimal demographic conditions. To help address this, we referred to a longitudinal study of bottlenose dolphins (*Tursiops truncatus*) around the Azores archipelago [24]. An open capture-recapture model yielded an estimate of adult survival of 0.970. Disturbance from whale watching, the major anthropogenic pressure to this population, appeared to cause no significant short-term reactions [45]. Consequently, we assumed this estimate was valid under optimal conditions and used it as input in our model. Finally, we obtained a distribution of λ*_max_*, incorporating uncertainty in AFR.

Subsequently we considered two plausible stock structure scenarii for common dolphin in the ENA: a single-stock scenario (supported by genetic markers; [10]) and a two-stock scenario (supported by ecological tracers; [11], [12]). Genetics and ecology studies are complementary for assessing marine mammal population structure. Genetics allow inference about breeding behaviours, pattern of gene flow and a population's demographic history (*i.e.* over several generations). Ecological studies provide data on the demography of natural populations, including the probability of an individual removed from one population being replaced by an individual from a nearby population, over time scales relevant to management (spanning between days to the lifespan of individuals) [46]. Incorporating available data on the population sizes of the neritic and oceanic stocks [14], [15], we estimated a PBR distribution for each stock in each scenario.

We compiled the results of the European, and French and English national onboard observer programs carried out under EU regulation 812/2004 to obtain annual bycatch for each stock (Table 1). We assumed that bycaught dolphins were part of the oceanic stock when the target species of the fishery was tuna and part of the neritic stock when the target species was a demersal fish like seabass. Bycatch varied greatly between years (see means and coefficients of variation in Table 1). They ranged from 357 to 1118 for the neritic stock, from 22 to 904 for the oceanic stock and from 379 to 1591 for the single stock.

**Table 1:** Annual bycatch estimates of common dolphins in the eastern North Atlantic compiled from European observer program PETRACET (Pelagic TRAwls and CETaceans) and French and English national observer programs under EU Regulation No 812/2004.

Year	Bycatch estimate			References
	Neritic stock	Oceanic stock	Single stock	
2003	439	-	439	[67]
	489*	133*	622*	[3]
	Total: 928	Total: 133	Total: 1061	
2004	145	-	145	[67]
	489*	133*	622*	[3]
	Total: 634	Total: 133	Total: 767	
2005	629	-	629	[5]
	489*	133*	622*	[3]
	Total: 1118	Total: 133	Total: 1251	
2006	1025	0	1025	[5]
	Total: 1025	Total: 0	Total: 1025	
2007	243	22	265	[68]
	114	-	114	[69]
	Total: 357	Total: 22	Total: 379	
2008	594	-	594	[70]
	396	0	396	[71]
	Total: 990	Total: 0	Total: 990	
2009	450	904	1354	[6]
	237	-	237	[72]
	Total: 687	Total: 904	Total: 1591	
Mean (CV)	820 (0.33)	189 (1.70)	1009 (0.37)	

*the bycatch estimates were the same for 2003, 2004 and 2005 because they were derived from the PETRACET program (Pelagic TRAwls and CETaceans) implemented from December 2003 to May 2005.

NB: We assumed that bycaught dolphins were part of the oceanic stock when the target species of the fishery was tuna and part of the neritic stock when the target species was a demersal fish like seabass.

For each stock, we generated 1000 bycatch numbers by picking at random 1 year from the 7 years considered and repeating this procedure 1000 times [20], [36]). Finally, for each stock scenario, we compared the generated bycatch distributions to the corresponding PBR distributions and compiled the risk that bycatch exceeded PBR.

### Ethics statements

The study was entirely based on data collected from cetacean carcasses found stranded along the French coasts and did not involve observation or experimentation on captive animals by any mean, nor did it rely on field observation of live animals.

The University of La Rochelle is the institution permanently in charge of running the French marine mammal stranding network under the decree of 10 November 2010, jointly taken by the Ministery in charge of the Environment and the Ministery in charge of Fisheries, regarding the use of biological data and samples collected on stranded marine mammals for scientific research and monitoring purposes.

## Results

### Available material

**Figure 2:**
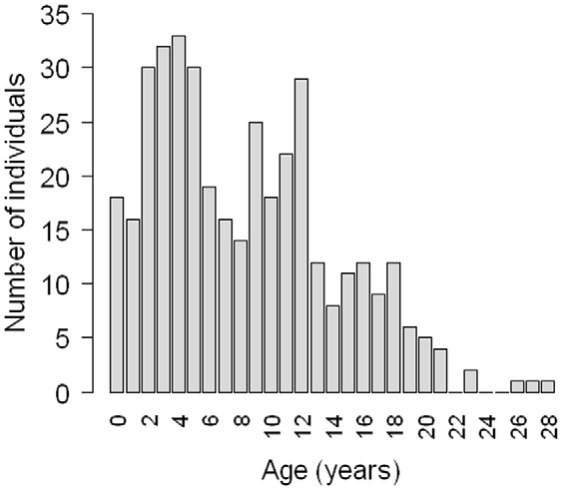
Age-at-death distribution for female common dolphins (sample size = 406).

The stranding dataset comprised 406 females of known age, of which at least 151 (37%) died in fishery operations. Their ages ranged from 0 to 28 years. The age-at-death distribution was multimodal with peaks in the age groups of juveniles (*i.e.* 2–5 years) and younger sexually mature adults (*i.e*. 9–12 years) (Fig. 2). This is quite different from what is expected under a stable age distribution where the greatest frequency is expected for yearlings, followed by juveniles and then adults. Bycatch affected age classes unequally. For example, between 2 and 5 years, about 50% of the individuals died following bycatch.

Reproductive state was known for 173 of the 406 aged females. Their ages ranged from 0 to 23 years. This sample comprised 78 immature and 95 mature females, of which 56 were categorized as resting, 25 as pregnant, 6 as lactating and 8 as pregnant and lactating.

### Reproductive parameters

**Figure 3:**
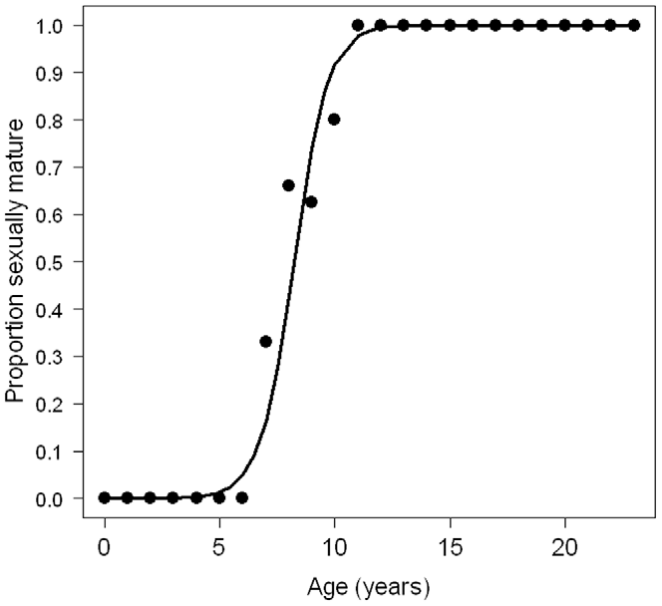
Logistic regression fitted to the proportion of mature females in each age class in order to estimate age at sexual maturity (sample size = 173).

All females were immature until 6 years and all females were mature after 11 years (Fig. 3, Table S1). The average ASM estimated from the logistic regression was 8.24 years (Fig. 3). The mean AFR was 9.23 (sd = 0.30) years.

**Figure 4:**
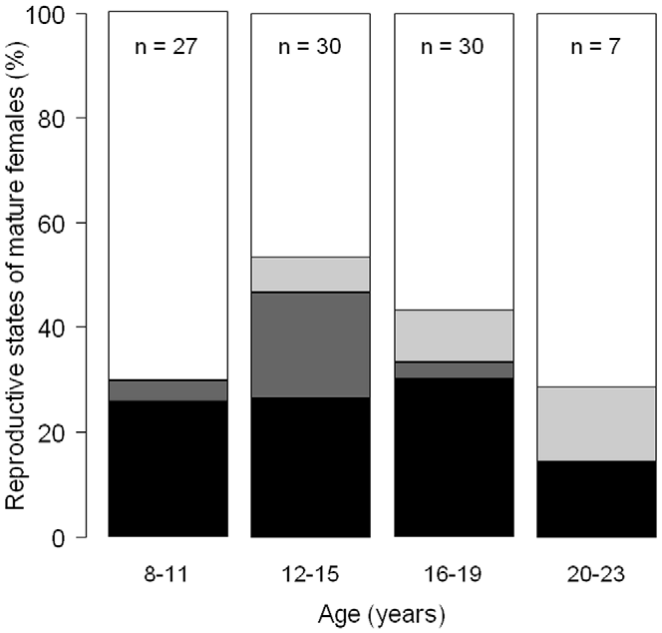
Proportion of reproductive states of mature females against age: pregnant (black), pregnant and lactating (dark grey), lactating (light grey) and resting (white). Samples sizes are shown at the top of the graph.

The youngest pregnant female was 8 years and the oldest was 20 years. Pregnancy rates were higher for 12–15 years old than for other age groups (Fig. 4). These rates corresponded to calving intervals of 3.33, 2.13, 3.03 and 7.14 years for the age groups 8–11, 12–15, 16–19 and 20–23 years respectively.

### Survival parameters

**Figure 5:**
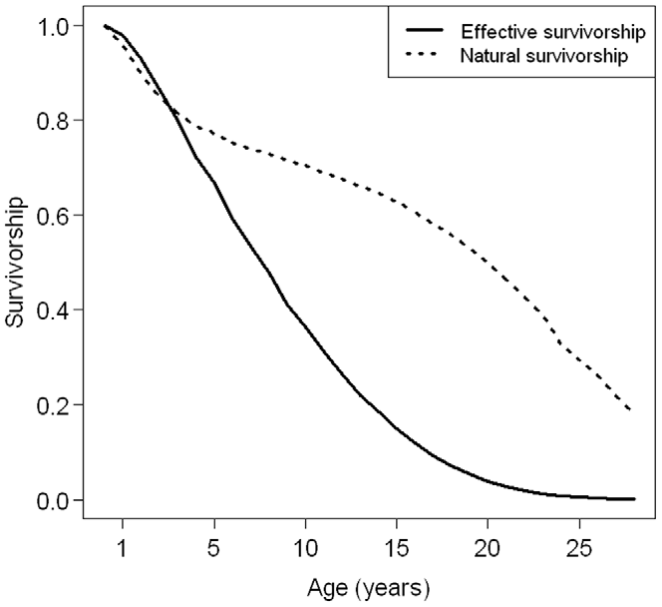
Effective survivorship for female common dolphins based on a maximum likehood fit of the Siler model. For indication, we also provided natural survivorship estimated with a similar maximum likelihood fit of the Siler model and based on a sample of bottlenose dolphins stranded in Florida (see [48]).

Effective survivorship based on maximum likelihood fit of the Siler model decreased at a constant rate through lifetime with values very close to 0 after 20 years (Fig. 5). The survivorship curve suggested that 90% of the females reach 2 years, only 60% reach 5 years and less than 30% reach 12 years. Effective age-specific survival (Table S2) appeared to be high at juvenile stage (especially for the first years of life) and very low at adult stage (respectively 0.92 and 0.84).

### Demographic modeling

#### Matrix model and deterministic population projections

**Figure 6:**
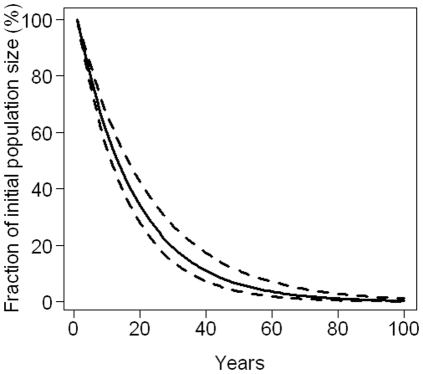
Deterministic projections of the common dolphin population over 100 years. Solid line: mean estimated effective growth rate. Dashed lines: 95% confidence intervals calculated from its mean and standard deviation.

The mean effective growth rate was 0.945 (sd = 5.10^−3^), suggesting that, currently, the population is decreasing at a mean rate of 5.5% per year. At this rate, the population would be divided by 5 in 30 years and extinct in 100 years (Fig. 6). Fertility, juvenile survival and adult survival elasticities were respectively 0.07, 0.6 and 0.3.

#### Demographic invariant method and risk analysis

**Figure 7:**
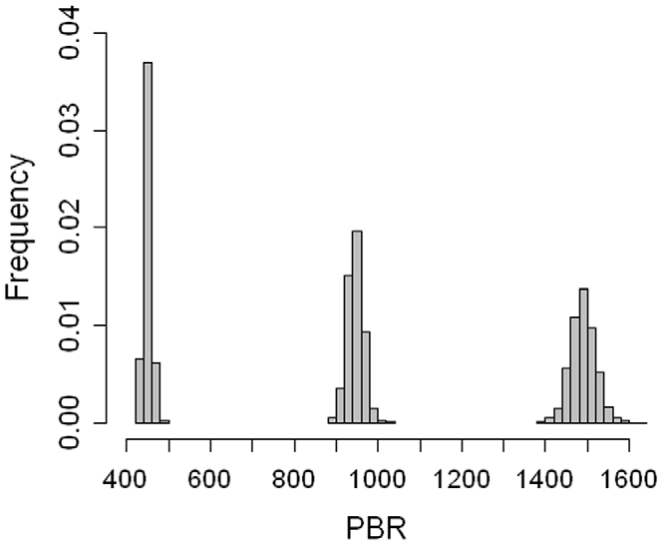
Frequency distribution of the Potential Biological Removal (PBR): for the neritic stock (mean = 450, sd = 9), the oceanic stock (mean = 945, sd = 19) and the single stock (mean = 1489, sd = 30).

The mean maximal growth rate was 1.045 (sd = 9.10^−4^), proposing that in optimal conditions the population would increase at a mean rate of 4.5% per year. Subsequently, the distributions yielded a mean PBR of 450 (sd = 9) common dolphins for the neritic stock, 945 (sd = 19) for the oceanic stock and 1489 (sd = 30) for the single stock (Fig. 7). Given the bycatch estimates in Table 1, under the two-stock scenario, the risk that bycatch exceeded PBR was 86.3% for the neritic stock and 0.4% for the oceanic stock. Under the single-stock scenario the risk was 14.4%.

## Discussion

The impact of bycatch on common dolphins in the eastern North Atlantic (ENA) was assessed using two approaches. Firstly, a matrix model built from strandings led to an effective growth rate lower than 1 (i.e., a declining population). Subsequently, deterministic projections suggested that the population would be reduced to 20% of its current size in 30 years (approximate lifespan of common dolphins). Secondly, the demographic invariant model provided further evidence for the deleterious impact of bycatch on the population. Considering our estimate of age at first reproduction and optimal adult survival from literature, we obtained a maximal growth rate greater than 1, indicating that in optimal conditions the population would increase at a rate of 4.5% per year. Subsequently, a risk analysis based on PBR suggested that the current bycatch level was likely too high for the neritic stock, when considering the two-stock scenario.

### Demographic parameters estimated from strandings

Our study relied on strandings, representing the only source of extensive demographic information on this population. Most studies estimating dolphin demographic parameters are based on longitudinal studies of live animals [24], autopsies of bycaught individuals [25] or individuals killed by directed fisheries [47]. Nevertheless, some authors used *postmortem* data gathered from stranded individuals to derive demographic parameters [26], [48] and conduct population viability analysis [16]. Historically, strandings provided extensive series of baseline measurements of mortality patterns, disease, toxic contaminant loads and other population indicators [49]. However, their use in demographic studies remained limited because of possible biases involved in sample collection.

Indeed, our stranding sample might be influenced by various factors. Firstly, mortality may not affect all age groups in all seasons equally. The majority of strandings occurred in winter which may reflect an increased bycatch mortality caused by the seasonal intensification of the bass pelagic trawl fishery [50]. Additionally the peak of juveniles in the age-at-death distribution (Fig. 2), also observed in other dolphin populations [51], reflects a bycatch selectivity possibly explained by a greater vulnerability of inexperienced individuals. Furthermore, meteorological factors (*e.g.* winds, tide, currents) determine how carcasses drift at sea and may represent a prevalent factor in stranding patterns [52]. Because of their greater distance from the coast, carcasses of dolphins living offshore are less likely to strand on the coast. Therefore our sample should be primarily composed of individuals living over the continental shelf. Once the carcass is stranded, reporting to the French stranding network depends on its persistence rate, detectability and observation effort [53].

However, there is no single source of cetacean demographic data free of any sampling bias. Photo-ID data suffer biases related to potential misidentification of individuals with temporary marks, underestimation of annual mortality arising from unequal periods between samples [54] or the inability to distinguish between permanent emigration and death leading to survival rate estimates that are negatively biased [24]. Analysis of age distribution from bycatch or directed catch also suffers important and uncontrollable biases such as segregation of individuals in the use of habitat or variation in vulnerability to bycatch [25].

We estimated demographic parameters on the basis of strandings collected over 30 years, not considering their possible variations over time; however, this source of uncertainty is reduced by the fact that as much as 66% of the data were collected from 2000 to 2006. The oldest female in our sample was 28 years old. This maximum age is comparable to previous studies in the eastern [26] or western North Atlantic [25] (respectively 29 and 25 years). Our estimates of reproductive parameters were close to those obtained from common dolphins stranded and bycaught in French waters (ASM: 8.55 years, pregnancy rates: 0.29) [26], and from common dolphins bycaught in swordfish driftnets in the western North Atlantic (ASM: 8.33 years, pregnancy rates: 0.25–0.33) [25]. In our estimation of pregnancy rates (ratio of pregnant females in the sample of mature females [25]), *intra utero* mortality is not considered and thus the production of calves is likely overestimated.

Matrix models require an estimate of age at first reproduction (AFR). Few AFR for delphinids are available in the literature. Some estimates are deduced from photo-ID studies. For example, AFR was established on the ages at which females of known age gave birth to their first viable calf [55]. However photo-ID studies should be long enough to provide accurate estimates of individual age and AFR. For the common dolphin population which is pelagic and widely distributed, we are unable to conduct photo-ID, thus we estimated AFR from autopsies. As we could not infer if females were primiparous or multiparous from our *postmortem* data, we assumed females conceive immediately after the attainment of sexual maturity [20], [36], [37], which is probably unrealistic for dolphins. For example, age at physical maturity is higher than ASM in bottlenose dolphins (respectively 13.09 and 10.64 years; [56]). Yet, as stated by Perrin and Donovan [37], this hypothesis is appropriate only if the first ovulation results in a birth. Therefore, AFR is likely underestimated. As a result, the reproductive parameters (AFR and pregnancy rates) used as inputs in our models may be optimistic, leading to a slight overestimation of population growth rate.

Few studies have estimated survivorship from age-at-death distributions derived from strandings [48]. Classical methods require that the population has a stable age distribution [57] which is not obvious for dolphins. Consequently, authors deduce survivorship from other large mammals, including terrestrial ungulates with similar life histories [20], [51]. This assumes that their survival schedule is representative of the dolphin population. Here, we estimated effective survivorship from the stranding age-at-death distribution which is non stable because it results from natural and anthropogenic mortality (at least 37% of the individuals died following bycatch). Consequently, our effective survivorship did not follow the expected pattern of natural survivorship (Fig. 5): a slight decrease during juvenile period, a slower decrease during adult period followed by a slight decrease [38]. We obtained very low effective survival for all ages, except for juveniles. This is probably the reason why juvenile survival elasticity was the greatest. These low estimates of survival may cause an underestimation of population growth rate.

### The management approach

We developed a management approach in which we focus on current population growth rate, given additional mortality and its theoretical growth rate under optimal conditions. It is applicable to other dolphin populations with consistent stranding datasets and provides indicators of population status and trajectory. Our models are based on easily estimated parameters and uncertainty is incorporated so that management decision can be facilitated in a timely manner [58]. Therefore, this approach may be valuable for managers.

We relied on management procedures broadly used in marine mammal conservation planning: PBR and population projections. There was also an attempt to implement the Catch Limit Algorithm of the International Whaling Commission [59] within a Bayesian framework to calculate bycatch limits for common dolphin in the ENA [60]. It necessitated time-series of population size and previous bycatch as inputs. The combination of data and model used were not informative about the population parameters of interest, notably population growth rate.

The PBR is a powerful tool for making management decisions when minimal information is available and for directing resources towards species of concern. The comparison of PBR estimates with current anthropogenic mortalities allows quick detection of potentially over-exploited populations [23]. Nevertheless, bycatch estimates from onboard observer programs under EU regulation No 812/2004 are likely to be severely underestimated for several reasons. Firstly, some EU countries have no dedicated observer programs and some countries like Spain, have no specific sampling programs to comply with the regulation, although they play a major role in the Bay of Biscay fisheries [61]. Secondly, currently most of the attention is being devoted to over 15 m vessels that form a minority of the fishing fleet. For example, gillnetters below 12 m, represent 84% of the French Atlantic fleet and 97% of UK fleet [9]. Then, within the fisheries surveyed, currently only 5 to 10% of the fishing effort is sampled and the bycatch observed in a minority of fishing trips is extrapolated to the total effort of the corresponding fleet segment. This provides unbiased estimates only if the observed trips are representative of all trips. This assumption can be violated if the behavior of fishermen is different when they carry an observer aboard. If bycatch vary predictably depending upon when and where the fishermen fish, vessels with observers can stay away from areas where they have experienced high incidental takes of dolphins [19]. Additionally, dolphin bycatch in pair-trawl fisheries is a sporadic event, but can involve a high number of individuals. For example, during the French onboard observer program in 2009, 94% of common dolphins bycaught in pair-trawlers were observed in only two trips, involving two pairs [6]. In contrast, none of the member states observed bycaught common dolphins in pair-trawlers in 2008. This results in highly fluctuating bycatch numbers as shown in Table 1. Another consequence of this variability is the high coefficients of variation (CVs) which make the estimates of little value.

Our analysis yielded a higher PBR for the single stock because of the higher size of this stock. Part of the difference in the assessments based on separate stocks rather than a single stock comes from the lower CV that results from combining different independent abundance estimates (the simple addition of the PBRs of the neritic and oceanic stocks gives 1395, a 6% reduction from the single stock PBR). We obtained a 86.3% risk that bycatch exceeded the PBR for the neritic stock, a close to zero risk for the oceanic stock under the two-stock scenario and a 14.4% risk under the single-stock scenario. As discussed in the previous paragraph, we believe that available bycatch estimates are highly biased downward and thus even the serious risk for the neritic stock is likely to be underestimated. Therefore the bycatch level would be unsustainable for the neritic population of the Bay of Biscay under the two-stock scenario. Additionally, considering the single-stock scenario, a mean bycatch of 1700 common dolphins per year with a CV of 0.15 would lead to a 80% risk that bycatch exceed PBR. Such underestimation of bycatch do not seem unlikely considering the imperfections in the fisheries observer programs and therefore a 80% risk would not be unrealistic under the single-stock scenario.

We also implemented deterministic projections built on a matrix model incorporating parameter uncertainty. We did not incorporate density dependence in our matrix (we assumed the population was below its carrying capacity), which may have lead to an underestimation of population growth rate [62]. Because of insufficient sample sizes, we did not allow between-year stochasticity [17]. The matrix model suggested an effective growth rate of −5.5% per year, on which we based our deterministic projections. Under the single stock scenario this effective growth rate would apply across the whole distribution of common dolphins. Under the two-stock scenario it would mainly apply to the neritic stock as stranding derived data mostly represent animals from shelf habitat.

Nevertheless, in both PBR and projections approaches, uncertainty did not alter the conclusion of a negative impact of bycatch on the population. We could have implemented a third approach, exploring different estimates of demographic parameters in the matrix (for example changing survival rates to represent increase or decrease of the bycatch level) and the resulting impact on the population growth rate. However, we are currently unable to associate a bycatch level to age-specific survival rates.

### Implications for management and research needs

Management strategies for cetacean populations need to be implemented in a timely manner because these species are characterized by a slow growth rate and a low resilience. Therefore, using only trends in abundance in management and conservation is a risky strategy for most cetacean species for which estimates of abundance are imprecise [58]. For example, it was noted that the endangered vaquita (*Phocoena sinus*) was likely to go extinct before a statistically significant trend could be determined [63]. Additionally, justifying the depleted status of dolphins killed by the tuna fishery in the eastern Tropical Pacific led to a delay of 14 years from the first abundance survey and a 23-year delay from the date of depletion [64]. On the contrary, our assessment of population growth rate based on demographic parameters estimated from stranded dolphins provided a quick detection of the deleterious impact of bycatch.

Our analysis revealed low fertility elasticity and high survival elasticity. This is typical of long-lived mammals that mature late and have few offspring [43]. Therefore the population will respond better to improved survival rates and management actions must be prioritized on reducing the level of bycatch in fisheries. Further, management actions should focus on the neritic stock for which the risk is the greatest. To reach the PBR level, the number of bycatch should be equal or less than 450 common dolphins. Then, effective conservation measures for our study population would be reducing bycatch to less than 50% of the current level in the neritic stock to reach PBR. This could be achieved by avoiding the use of fishing gears that cause dolphin mortality, in areas where dolphins are present, and encouragement of more selective fishing methods.

The collection of teeth and reproductive tracts from stranded dolphins should be carried on to provide additional material to refine demographic estimates. Census must be continued in the ENA and estimation of abundance must be improved to provide precise estimates of population size that have a strong weight in PBR calculation. Fisheries monitoring by all member states must be improved and expanded to all fleet segments to provide more robust bycatch estimates. Sampling strategy for vessels under 15 m needs to be established, taking into account the specific problems with monitoring such vessels [61]. To do this, the implementation of Rapid Bycatch Assessment (RBA, [65]), based on fishermen interviews, could be an appropriate methodology. Moreover, attempts at estimating the proportion of bycatch and natural mortality in the overall mortality on the basis of stranded individuals should be carried out. This could be confronted with bycatch mortality estimated from observer programs (percentage of bycaught individuals in the overall population).

A striking outcome of our study is the pessimistic management diagnostic considering two stocks instead of a single stock in the eastern North Atlantic. In 2010, the sub committee on Small Cetaceans of the International Whaling Commission stated that the lack of detection of genetic structure does not necessarily mean that structure is absent [8]. Therefore, we believe that the definition of management units for common dolphin in the ENA is a major question for addressing the issue of bycatch. As advised by the International Whaling Commission [8] and ASCOBANS/HELCOM [66], the use of additional markers, including markers reflecting the ecological time scales should be encouraged, with the collection of large samples from all sex/age classes and over a large geographic scale. All this information is necessary to assess the impact of bycatch and develop adequate conservation measures.

## Supporting Information

Table S1 **Detailed reproductive states of females for each age class.**
(DOCX)

Table S2 **Age-specific survival based on common dolphin ages-at-death (effective survival).** In addition, natural survival estimation from stranded common dolphins in Florida [48] is provided.
(DOC)
