# Supplementary material for: Assessing the Impact of Bycatch on Dolphin Populations: The Case of the Common Dolphin in the Eastern North Atlantic
Source: PLoS One. 2012 Feb 29;7(2):e32615. doi: 10.1371/journal.pone.0032615 (PMC3290591; doi:10.1371/journal.pone.0032615)
Supplement: Table S1 — Detailed reproductive states of females for each age class. (DOCX) [file pone.0032615.s001.docx]

**Table S1**

| Age (years) | Immature females | | Mature females | | | | | | | | | | Total number of females |
| --- | --- | --- | --- | --- | --- | --- | --- | --- | --- | --- | --- | --- | --- |
|  |  |  | Resting | | Pregnant | | Pregnant and lactating | | Lactating | | All mature | |  |
|  | number | % | number | % | number | % | number | % | number | % | number | % |  |
| 0 | 10 | 100 | 0 | 0 | 0 | 0 | 0 | 0 | 0 | 0 | 0 | 0 | 10 |
| 1 | 10 | 100 | 0 | 0 | 0 | 0 | 0 | 0 | 0 | 0 | 0 | 0 | 10 |
| 2 | 9 | 100 | 0 | 0 | 0 | 0 | 0 | 0 | 0 | 0 | 0 | 0 | 9 |
| 3 | 15 | 100 | 0 | 0 | 0 | 0 | 0 | 0 | 0 | 0 | 0 | 0 | 15 |
| 4 | 16 | 100 | 0 | 0 | 0 | 0 | 0 | 0 | 0 | 0 | 0 | 0 | 16 |
| 5 | 7 | 100 | 0 | 0 | 0 | 0 | 0 | 0 | 0 | 0 | 0 | 0 | 7 |
| 6 | 3 | 100 | 0 | 0 | 0 | 0 | 0 | 0 | 0 | 0 | 0 | 0 | 3 |
| 7 | 2 | 66.7 | 1 | 33.3 | 0 | 0 | 0 | 0 | 0 | 0 | 0 | 33.3 | 3 |
| 8 | 2 | 33.3 | 3 | 50 | 0 | 0 | 1 | 16.7 | 0 | 0 | 4 | 66.7 | 6 |
| 9 | 3 | 37.5 | 5 | 62.5 | 0 | 0 | 0 | 0 | 0 | 0 | 5 | 62.5 | 8 |
| 10 | 1 | 20 | 2 | 40 | 2 | 40 | 0 | 0 | 0 | 0 | 4 | 80 | 5 |
| 11 | 0 | 0 | 9 | 64.3 | 5 | 35.7 | 0 | 0 | 0 | 0 | 14 | 100 | 14 |
| 12 | 0 | 0 | 4 | 36.4 | 3 | 27.2 | 4 | 36.4 | 0 | 0 | 11 | 100 | 11 |
| 13 | 0 | 0 | 6 | 75 | 0 | 0 | 1 | 12.5 | 1 | 12.5 | 8 | 100 | 8 |
| 14 | 0 | 0 | 1 | 20 | 3 | 60 | 1 | 20 | 0 | 0 | 5 | 100 | 5 |
| 15 | 0 | 0 | 3 | 50 | 2 | 33.3 | 0 | 0 | 1 | 16.7 | 6 | 100 | 6 |
| 16 | 0 | 0 | 6 | 66.7 | 3 | 33.3 | 0 | 0 | 0 | 0 | 9 | 100 | 9 |
| 17 | 0 | 0 | 4 | 66.7 | 1 | 16.6 | 0 | 0 | 1 | 16.7 | 6 | 100 | 6 |
| 18 | 0 | 0 | 4 | 40 | 4 | 40 | 1 | 10 | 1 | 10 | 10 | 100 | 10 |
| 19 | 0 | 0 | 3 | 60 | 1 | 20 | 0 | 0 | 1 | 20 | 5 | 100 | 5 |
| 20 | 0 | 0 | 2 | 66.7 | 1 | 33.3 | 0 | 0 | 0 | 0 | 3 | 100 | 3 |
| 21 | 0 | 0 | 2 | 66.7 | 0 | 0 | 0 | 0 | 1 | 33.3 | 3 | 100 | 3 |
| 22 | 0 | 0 | 0 | 0 | 0 | 0 | 0 | 0 | 0 | 0 | 0 | 0 | 0 |
| 23 | 0 | 0 | 1 | 100 | 0 | 0 | 0 | 0 | 0 | 0 | 1 | 100 | 1 |
