# Supplementary material for: Assessing the Impact of Bycatch on Dolphin Populations: The Case of the Common Dolphin in the Eastern North Atlantic
Source: PLoS One. 2012 Feb 29;7(2):e32615. doi: 10.1371/journal.pone.0032615 (PMC3290591; doi:10.1371/journal.pone.0032615)
Supplement: Table S2 — Age-specific survival based on common dolphin ages-at-death (effective survival). In addition, natural survival estimation from stranded common dolphins in Florida [48] is provided. (DOC) [file pone.0032615.s002.doc]

**Table S2**

| Age | Age-specific survival | |
| --- | --- | --- |
|  | Effective | Natural |
| 0 | 0.9800710 | 0.9581310 |
| 1 | 0.9488192 | 0.9361576 |
| 2 | 0.9284272 | 0.9457996 |
| 3 | 0.9263321 | 0.9625345 |
| 4 | 0.9033752 | 0.9650799 |
| 5 | 0.9222414 | 0.9788208 |
| 6 | 0.8912233 | 0.9766135 |
| 7 | 0.8984850 | 0.9822780 |
| 8 | 0.8927954 | 0.9837579 |
| 9 | 0.8649003 | 0.9812611 |
| 10 | 0.8812737 | 0.9845536 |
| 11 | 0.8551934 | 0.9811110 |
| 12 | 0.8535695 | 0.9806654 |
| 13 | 0.8310875 | 0.9765912 |
| 14 | 0.8370149 | 0.9766285 |
| 15 | 0.8131022 | 0.9713917 |
| 16 | 0.7910553 | 0.9657303 |
| 17 | 0.7785496 | 0.9613486 |
| 18 | 0.7727067 | 0.9581550 |
| 19 | 0.7431299 | 0.9491963 |
| 20 | 0.7212508 | 0.9411771 |
| 21 | 0.6974241 | 0.9318995 |
| 22 | 0.7161360 | 0.9186220 |
| 23 | 0.6036172 | 0.9114129 |
| 24 | 0.5977226 | 0.8379828 |
| 25 | 0.6634916 | 0.9033291 |
| 26 | 0.4928219 | 0.8929837 |
| 27 | 0.5116942 | 0.8389250 |
| 28 | 0.4741509 | 0.8150377 |
